# A Transmission Model for the Ecology of an Avian Blood Parasite in a Temperate Ecosystem

**DOI:** 10.1371/journal.pone.0076126

**Published:** 2013-09-20

**Authors:** Courtney C. Murdock, Johannes Foufopoulos, Carl P. Simon

**Affiliations:** 1 School of Natural Resources & Program in the Environment, University of Michigan, Ann Arbor, Michigan, United States of America; 2 Center for Infectious Disease Dynamics, Department of Entomology, Pennsylvania State University, University Park, Pennsylvania, United States of America; 3 Ford School of Public Policy & Center for the Study of Complex Systems, Department of Mathematics, University of Michigan, Ann Arbor, Michigan, United States of America; Monash University, Australia

## Abstract

Most of our knowledge about avian haemosporidian parasites comes from the Hawaiian archipelago, where recently introduced *Plasmodium* *relictum* has contributed to the extinction of many endemic avian species. While the ecology of invasive malaria is reasonably understood, the ecology of endemic haemosporidian infection in mainland systems is poorly understood, even though it is the rule rather than the exception. We develop a mathematical model to explore and identify the ecological factors that most influence transmission of the common avian parasite, *Leucocytozoonfringillinarum* (Apicomplexa). The model was parameterized from White-crowned Sparrow ( *Zonotrichia* *leucophrys* ) and *S. silvestre* */ craigi* black fly populations breeding in an alpine ecosystem. We identify and examine the importance of altricial nestlings, the seasonal relapse of infected birds for parasite persistence across breeding seasons, and potential impacts of seasonal changes in black fly emergence on parasite prevalence in a high elevation temperate system. We also use the model to identify and estimate the parameters most influencing transmission dynamics. Our analysis found that relapse of adult birds and young of the year birds were crucial for parasite persistence across multiple seasons. However, distinguishing between nude nestlings and feathered young of the year was unnecessary. Finally, due to model sensitivity to many black fly parameters, parasite prevalence and sparrow recruitment may be most affected by seasonal changes in environmental temperature driving shifts in black fly emergence and gonotrophic cycles.

## Introduction

While locally transmitted human malaria in the United States was eradicated around 1950, avian blood parasites are endemic throughout the USA and most of the world [1]. In fact, avian malaria imposes strong threats to persistence of immunologically naïve island native bird populations [2]. The co-introduction of *Plasmodium* *relictum* and the mosquito *Culex* *quinquefaciatus* in Hawaii in 1826, in conjunction with habitat degradation and invasive predators, led to the endangerment and extinction of many endemic bird species [3-7]. Although the Hawaiian epidemic has been fairly well investigated and modeled [8-10], there is surprisingly little theoretical work (either conceptual or analytic) describing transmission dynamics in mainland systems (exceptions are [11-13]). Continental birds in the U.S.A. share a longer history with avian blood parasites, are infected with a greater diversity of these parasites, and such infection is the rule rather than the exception [1]. In this paper we develop and analyze a model for the transmission of a related blood parasite in a songbird population breeding in a temperate USA ecosystem.

Human malaria is caused by four species of parasite in the genus *Plasmodium* . However, in birds, there are over 200 morphologically defined taxa of avian blood parasites spanning three genera *Plasmodium* , *Haemoproteus*, and *Leucocytozoon* (Apicomplexa: Haemosporida) [1]. Parasite species in these genera are transmitted between avian hosts through the bite of a fly vector (Order Diptera: *Plasmodium* , Culicidae; *Haemoproteus*, Ceratopogonidae and Hippoboscidae; *Leucocytozoon,* Simuliidae). These species have broad global distributions and can infect a wide diversity of avian families [1]. Much empirical work has been done on the ecology and fitness effects of malarial parasites (e.g. [1,3,14]). Yet, our general knowledge across different groups of haemosporidians is unevenly distributed, with most attention devoted toward human malaria and the genus *Plasmodium* [1]. Nonetheless, infections with avian haemosporidians have been characterized by high population prevalence and severe pathology in the acute phase [14-16]. Although acutely infected young birds can succumb to haemosporidians, surviving adults typically carry chronic, *sublethal* infections that can reduce breeding success [17-19], body condition [20], immunity [21], and survival [22,23].

The density of parasite stages in the blood (parasitemia) changes dynamically throughout the course of an infection and is positively correlated to infectivity to the dipteran vector [24,25]. Upon infection, a bird enters the acute phase of the infection characterized by an initial spike in blood stage parasitemia; this phase can range from one week to several months depending on the parasite species, vertebrate host, and environmental factors. At the end of the acute infection, parasitemia (and infectiousness) decreases (to roughly 1-3 gametocytes per 10,000 red blood cells), and birds enter a chronic phase of infection that also varies in duration [1]. Upon exiting the chronic phase of infection, the bird enters a latent stage of infection (no longer infectious), in which parasites disappear from the peripheral blood but persist in non-circulating tissues, such as the internal organs [1].

Here we develop a model for haemosporidian transmission in a songbird population breeding in a temperate, strongly seasonal ecosystem. The purpose of this model is to define the key parameters for transmission ecology in this system, and to examine the role of seasonal relapse and nude susceptible nestlings in parasite persistence across seasons. For many blood parasite species in temperate regions, the majority of birds with latent infections (birds infected during the prior breeding season) return to the breeding grounds and experience an increase in parasite blood stages - a relapse (e.g. [1,26-32]). This relapse occurs prior to the emergence of biting dipteran vectors [29]. This elevation of parasitemia is initiated by seasonal increases in sexual hormones and corticosterone in early spring; these hormones stimulate parasite emergence out of deep tissues in latently infected birds [33,34]. Seasonal relapses are considered to be an adaptive strategy of the parasite to time transmission effectively on temperate breeding grounds as susceptible, first year birds are being introduced into the system [12].

**Figure 1:**
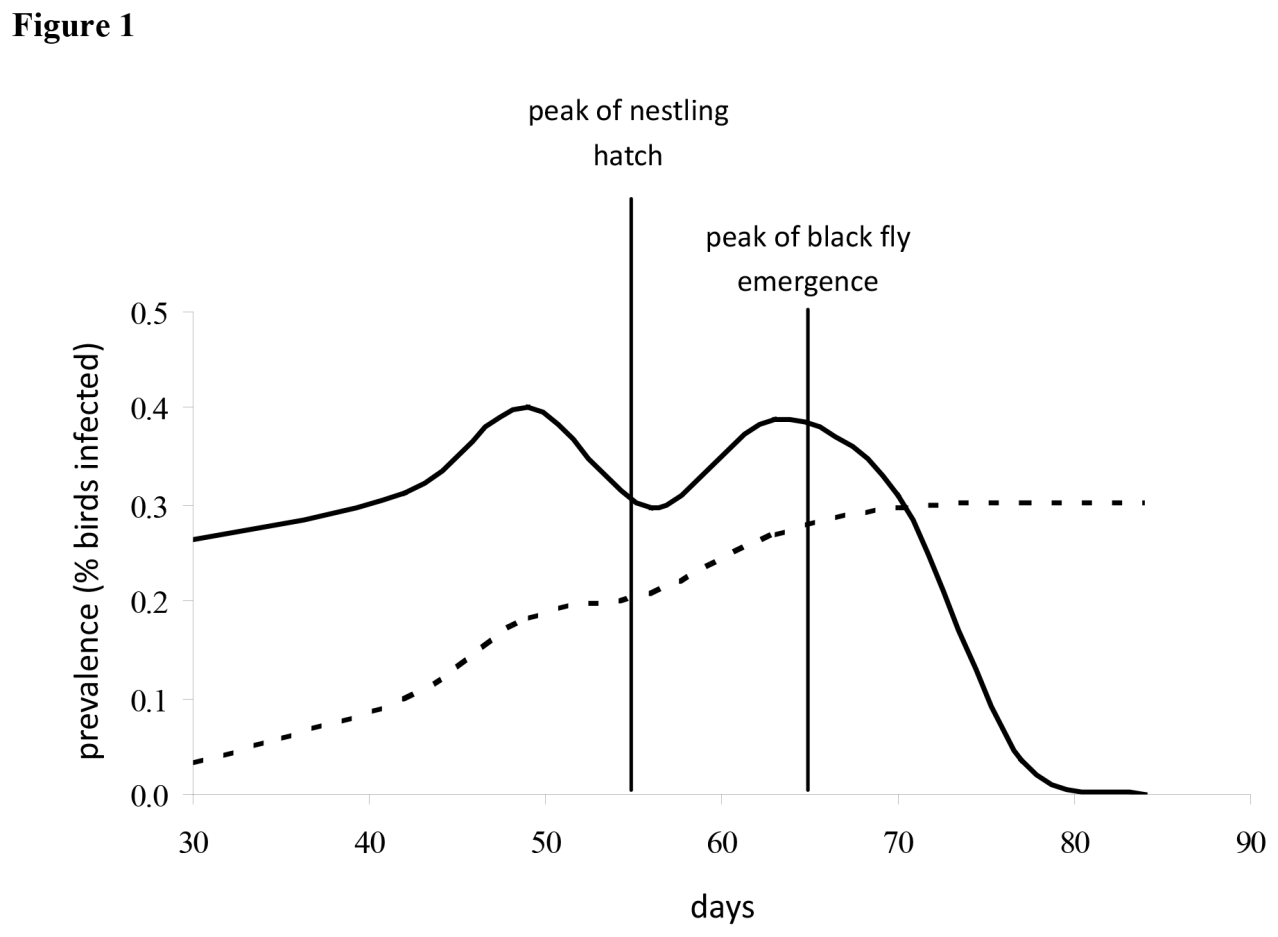
Parasite prevalence in the sparrow population peaks twice during the breeding season. In 2005, parasite prevalence of *L*. *fringillinarum* (solid line; likely comprising acute, relapsing, and chronic infections) peaks twice throughout the breeding season (day 30 equals May 30^th^). The first peak corresponds prior to the date of peak nestling hatching and black fly emergence (vertical dotted lines). Based on when they occur during the season, the first and second prevalence peaks most likely correspond to relapsing infectious adult and newly infected first year birds, respectively. Cumulative parasite prevalence indicates that the end of the season parasite prevalence in the sparrow population is 30% in 2005 (dashed line).

The parasitemia associated with the relapse is lower, but of longer duration than *acute* infections [29]. As compared to the *chronic* stage of infection, which occurs following an acute infection, parasitemia in the bloodstream and transmission of parasites from relapsing hosts to dipteran vectors increases during the spring relapse. As a result of spring relapse, two peaks in parasite prevalence often occur throughout the breeding season; the first corresponds to relapsing infectious adult birds, the second corresponds to newly infected young of the year birds (Figure 1, [1,12,29]).

## Model

### Ethics statement

Field work for this study (Text S1) was carried out in strict accordance with the recommendations and approval of the University Committee on Use and Care of Animals at the University of Michigan (Permit Number: 09077) and the Rocky Mountain Biological Laboratory’s Research Committee. All birds were immediately removed from shaded mist nets and ground traps, handled for less than 10 minutes, and all efforts were made to minimize suffering. The amount of blood collected for parasite and condition assays was very small relative to an individual’s body mass and well below veterinary guidelines. We obtained both a federal banding permit (#2328) and a Colorado Fish and Wildlife Service License (09TRb1094) for field work associated with this study.

### The models

The models consider the transmission of *Leucocytozoon* in a passerine population breeding in an alpine ecosystem. The models are relatively complex because they must include three different interacting levels: birds, flies and parasites, with the bird population age-structured and stage-structured. Because these models represent a first attempt in modeling transmission dynamics in this system, we made some simplifying assumptions to keep these analytic models tractable. Our models work with a single generic avian species, a single vector, and a single parasite. Further, due to the paucity of data on transmission dynamics among sparrows in their wintering grounds (desert habitats of Northern Mexico), we are assuming that no transmission occurs overwinter. The arid conditions and warm climates in these systems make the absence of Simuliid vectors almost certain. Future, models will begin to relax these assumptions to incorporate additional complexity. For example, multiple bird hosts and vector species with varying competence levels, competition among parasite species in mixed infections, as well as vector feeding preferences for different avian species are most likely important in this system. However, this task will require very different, more complex models than those discussed in this paper. The model presented here lays the foundation for more complex models in the future.

Transmission in the models depends upon the interaction between an age-structured (first year and adult) and stage-structured (not feathered and feathered) bird reservoir populations and a sympatric black fly vector population. We had the following objectives: 1) build a basic model to describe this system, 2) compare the model with prevalence data in the bird population, 3) use the model to estimate unknown parameters, and 4) run a sensitivity analysis to determine the parameters that most affect transmission dynamics. The following questions were addressed with these models: 1) what parameters most impact transmission and parasite prevalence in this system, 2) do nude nestlings affect overall transmission dynamics, and 3) is seasonal relapse of latently infected individuals important for maintaining transmission in this system?

The models are a variation of the classic Susceptible (*S*), Exposed (*E*), Infectious (*I*) model [35] for the transmission of *Leucocytozoonfringillinarum* , and were parameterized with demographic and infection prevalence data collected from a Mountain White-crowned Sparrow ( *Zonotrichia* *leucophrys* *oriantha*) host population and a local black fly (*Simulium silvestre/S. craigi*) vector population breeding on three field sites (but see Text S1 for additional potential avian hosts and black fly vectors). All field sites were within the vicinity of the Rocky Mountain Biological Laboratory (RMBL), Gunnison County, Colorado, U.S.A. (see Text S1 for details). Because of their high elevation (3,000 m asl) the study plots are covered with deep snow until late spring when migrating White-crowned Sparrow males occupy territories in the valley bottom. As the snowfields melt, the birds commence breeding activities while overwintering black fly populations complete their development and emerge to feed on the resident vertebrates.

We chose White-crowned Sparrows to model blood parasite transmission because a substantial foundation of baseline knowledge exists for this species’ habitat preferences, food habits, behavior, breeding, physiology, and predators (for reviews [36,37]). The most prevalent blood parasite species within the sparrow population is *L* *. fringillinarum* [38] and it has been shown to exhibit seasonal relapses [32]. We chose *S* *. silvestre* / *S* *. craigi* (these two black fly species were combined into one morphospecies due to logistical difficulties in distinguishing them through morphological characteristics alone) as a potential vector because it is: 1) the most abundant ornithophilic black fly species at these field sites, 2) present throughout the majority of the breeding season (June-August), 3) found to harbor a large diversity of *Leucocytozoon* spp. [38], and 4) is linked to transmission of *L* *. fringillinarum* in other systems [39]. Additionally, unlike many mosquito species that transmit *Plasmodium* parasites, *S. silvestre* */ craigi* obtain their required bloodmeal through only *one* bite (versus multiple bites) to complete a reproductive cycle (Peter Adler, personal communication).

### Single season model

We constructed the basic model to consist of a single transmission season (see Text S2 for equations and basic model) and included modules for both the bird (*B*) and black fly (*F*) populations. The bird population is further structured into an adult bird module (*A*) and a first year bird module (*J*). In the first year bird module, we separated nude nestlings from feathered first year birds because we assume that nude nestlings will be more accessible to biting vectors when encountered due to the lack of feathers. When birds return to the breeding grounds in early spring, they enter the adult bird module (*A*) as either susceptible (*S*_*A*_) or exposed (*E*_*A*_, infected but not infectious). Susceptible adults remain susceptible throughout the entire transmission season or become exposed with a probability of *b*_*F*_ if bitten by an infectious black fly vector. Exposed adults become acutely infectious (*I*_*A*_) at a rate (*λ*_*B*_). Acutely infectious adults transition to chronically infectious adults (*CI*_*A*_) at a rate (*δ*). All adult birds experience a background natural mortality rate (*d*_*2*_); acutely infectious adults experience a parasite-induced mortality rate *d*_*3*_.

Adult birds produce nestlings (*N*) that hatch in a time-dependent manner throughout the season (May-August) and enter the first year bird module as susceptible, nude nestlings (*S*_*N*_, Text S2). The nestling birth function was parameterized from empirical nest-monitoring data (Table 1; Figure 1A in Text S1) and was closely approximated by an exponential / quadratic curve in the model (Text S1). Nude nestlings enter the first year bird class (*J*) in a variety of states; they either become susceptible feathered first year birds (*S*_*J*_) at a rate *X*, or if bitten by an infectious black fly vector become exposed feathered first year birds (*E*_*J*_) with a probability of *b*_*N*_. Susceptible feathered first year birds can either remain susceptible throughout their first season, or if bitten by an infectious black fly vector, become exposed feathered first year birds at rate *b*_*F*_. The model also assumes that the prepatent period (time to infectiousness) is the same for both first year and adult birds infected for the first time. Thus, exposed feathered first year birds become acutely infectious (*I*_*J*_) at rate *λ*_*B*_, after which they enter the chronically infectious stage (*CI*_*J*_) at rate δ. All nude nestlings experience a higher background death rate (*d*_*1*_) than feathered first year and adult birds (*d*_*2*_), while acutely infectious first year birds experience a parasite-induced death rate *d*_*3*_.

**Table 1:** Parameter descriptions and initial values.

**Bird Parameters**	**Average Value**	**Definition**	**Reference**
b_N_	0.900	transmission probability from infectious black fly to susceptible nestling	8, 80
b_F_	0.300	transmission probability from infectious black fly to susceptible feathered bird	81
λ_B_	0.182 day ^-1^	rate an exposed bird becomes acutely infectious	1, 82
δ	0.095 day ^-1^	rate an acutely infectious bird becomes chronically infectious	1, 82
δ_R_	0.154 day ^-1^	rate a relapsing infectious bird becomes latently infected (overwintering bird)	29
σ_J_	0.230 day ^-1^	rate a chronically infectious YOY bird becomes latently infected (overwintering bird)	MP
σ_A_	0.230 day ^-1^	rate a chronically infectious adult birds become latently infected (overwintering bird)	MP
γ_J_	15 / (135 days-q_B_)	rate a susceptible YOY bird becomes an overwintering, susceptible bird	MP
γ_A_	0.185 day ^-1^	rate a susceptible adult bird becomes an overwintering, susceptible bird	MP
*X*	0.167 day ^-1^	rate nude nestlings acquire feathers	JF unpublished
d_1_	0.017 day ^-1^	natural death rate of YOY nestlings	JF unpublished, 36, 37
d_2_	0.000616 day ^-1^	natural death rate of feathered YOY and adults	36, 37
d_3_	0.00104 day ^-1^	death rate of acutely infectious birds	1, 27
A_B_	0.148 YOY female ^-1^ day ^-1^	the peak number of nestlings hatching per day	JF unpublished, 37
c_B_	200	support for the nestling hatch function	JF unpublished, 37
q_B_	57 day	the date when the peak number of nestlings hatch	JF unpublished
y_J_	1.00	proportion of latently infected YOY that return to breed as relapsing infectious adults	?
y_A_	1.00	proportion of latently infected adults that return to breed as relapsing infectious adults	?
x_J_	0.20	proportion of YOY birds surviving overwinter to return to breed	37, 83
x_A_	0.80	proportion of adult birds surviving overwinter to return to breed	37
susceptible birds	1, 359 day	number of days oven fills & cooks for the susceptible juvenile & adult oven	MP
latently infectious birds	1, 359 day	number of days oven fills & cooks for the latently infectious juvenile & adult oven	MP
**Black Fly Parameters**			
r	0.133 bites day ^-1^	number of bites a black fly takes per day	39, 84, 85
b_A_	0.500	transmission probability from an acutely infectious bird to susceptible fly	86
b_R_	0.300	transmission probability from a relapsing infectious bird to susceptible fly	?
b_C_	0.050	transmission probability from a chronically infectious bird to susceptible fly	?
λ_F_	0.143 day ^-1^	rate of an exposed black fly becomes infectious	1, 82
σ_F_	0.055 day ^-1^	rate of an infectious black fly flowing through each chain in the infectious class	MP
d_4_	0.17	natural death rate of black flies	38
A_F_	70 flies ^-1^ day ^-1^	the peak number of black flies emerging per day	CM field data
c_F_	600	support for the black fly emergence function	CM field data
q_F_	66 day	the date the peak number of black flies emerge	CM field data

? = data unknown

MP = model programmed parameters that are designed to ensure all birds and black flies leave the summer season at the appropriate time, control for differential overwinter mortality between first year and adult birds, and to manage the cook times associated with the overwinter ovens.

JF = Johannes Foufopoulos

CM = Courtney Murdock

The third module represents the vector population of black flies (*F*). Black flies emerge from their aquatic habitats in a time-dependent manner and enter the module as susceptible black flies (*S*_*F*_). The black fly emergence function was parameterized from empirical insect data (Table 1; Figure 1B in Text S1) and was approximated by an exponential / quadratic curve in the model (Text S1). Upon emergence, black flies either remain susceptible throughout the breeding season or become exposed (*E*_*F*_) with a probability of *b*_*A*_, *b*_*R*_, or *b*_*C*_ (summary parameter *P*; Text S2) if they have fed on an acutely, a relapsing, or a chronically infectious bird, respectively. Exposed black flies then become infectious (*I*_*F*_) at a rate *λ*_*F*_.

All susceptible, exposed, and infectious black flies are assumed in this model to experience a background mortality rate (*d*_*4*_). Additionally, black fly adults do not survive the winter on these sites, but instead overwinter in the egg stage in high alpine habitats [39]. In the spring, larvae hatch, progress through seven instars of development, and molt into pupae [39-41] (not modeled in this study). Adults then begin to emerge around the first week of June (Figure 1B in Text S1). We are also assuming in this model that black fly adult females require one bloodmeal per reproductive cycle, they take only one bite to produce a clutch of eggs, and they only feed on avian hosts prior to egg laying (Peter Adler, personal communication and [39]). Thus, the contact rate (r) between birds and black flies in all modules is determined by the reproductive schedule and lifespan (maximum 30 days) of black flies throughout the transmission season.

### Extensions of the single season model

**Figure 2:**
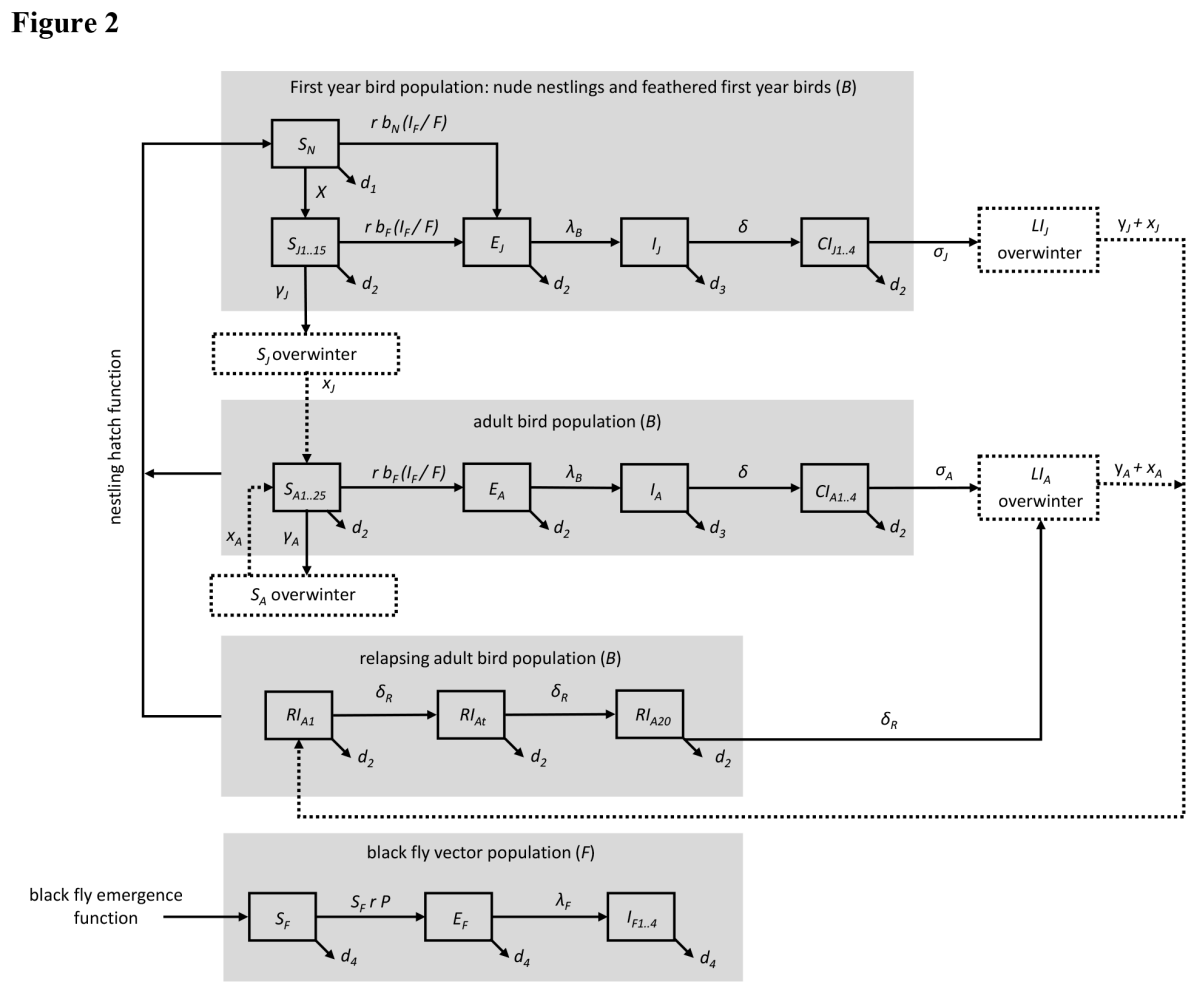
Extensions of the simple model. The multi-year model adds a relapsing infectious adult bird compartment and *ovens* (dashed compartments), which represent overwintering birds in various states (adult or first year birds that are susceptible or latently infected). Arrows denote flows of individuals entering or leaving compartments within each module over time. ***B*** and ***F*** in the module headings represent the total number of individuals in the bird and black fly vector populations, respectively. The first one or two letters of each compartment label corresponds to the infection status of individuals entering or leaving that compartment (***S*** = susceptible, ***E*** = exposed, ***I*** = acutely infectious, ***CI*** = chronically infectious, ***RI*** = relapsing infectious, and LI = latently infected). Subscripts in each compartment label corresponds to the population the module represents (***N*** = nude nestling and ***J*** = feathered first year birds, ***A*** = adult bird population, and ***F*** = black fly vector population). In the multi-year model model, ***P*** is now equal to the sum of [*b*_*A*_ (*I*_*A*_ + *I*_*J*_) + *b*_*C*_ (*CI*_*A*_ + *CI*_*J*_) + *b*_*R*_ *RI*_*A*_] / *B*. To ensure approximately all susceptible, chronically infectious, and relapsing infectious birds transition into the corresponding overwinter oven, we added chains or compartments to these stages (chains = *S*_*J*_: 15; *S*_*A*_: 25; *CI*_*J*_ and *CI*_*A*_: 4; *RI*_*A*_: 20). To ensure all black flies were dead by the end of the breeding season, we added four chains to the infectious black fly class (I_F_). Initial conditions for the compartments in the modules are the following: 100 sparrows begin as susceptible adults (S_A_) and 100 sparrows enter as relapsing infectious adults (RI_A_). All other compartments initially begin with zero individuals.

To examine the role of relapse for seasonal parasite persistence, we extended the basic transmission model to run for multiple seasons (Figure 2; for associated equations see Text S3). As a result, in both the adult and first year bird modules, four overwinter compartments were added for birds that have migrated from the summer breeding grounds to their overwintering sites: susceptible adult (*S*_*A*_) and first year birds (*S*_*J*_), and latently infected adult (*LI*_*A*_) and first year (*LI*_*J*_) birds (chronically infectious adults and first year birds become latently infected overwinter at rates *σ*_*A*_ and *σ*_*J*_, respectively). In the adult bird module, we added a relapsing infectious adult compartment (*RI*_*A*_) because *L* *. fringillinarum* can cause spring relapses (e.g. [11,27,31]). Thus, a fraction (initially set to one) of latently infected adult and first year birds that return the subsequent breeding season enter the adult bird module as relapsing infectious adults. To account for overwinter mortality, 80% and 20% of the first year and adult bird populations, respectively, die over the winter [37].

The structure and parameter choices of the differential equations lead to rather gradual transitions between stages, while the data suggest more abrupt changes for specific stages. To account for the more abrupt changes suggested in the field data, especially for the timing of fall migration, we added chains in the susceptible, chronically infectious, and relapsing infectious bird compartments so that all birds would migrate at the appropriate time of season to their wintering grounds. We also added chains to the infectious black fly compartment so that all flies die at the end of the summer season [42,43].

### Parameter selection and model assumptions

The final model contains 23 fixed parameters and 2 time-dependent functions (Text S4). It also includes four overwinter periods for susceptible and latently infected first year and adult birds. When possible, we selected parameter values and initial conditions from the literature and from field data on sparrow and black fly populations collected from sites in Colorado.

The modeling process always involves some simplifying assumptions. These models made the following assumptions. 1) We did not incorporate a recovered class in this model for either the bird host or black fly vector. Substantial evidence demonstrates that most bird species do not develop complete immunity to malarial parasites and remain chronically infected throughout their lives [1,14]. Additionally, there is no evidence that biting vectors clear sporozoites from the salivary glands after invasion [1], suggesting that infectious flies remain infectious throughout the duration of their relatively short lifespans (longevity as adults is approximately seven to 30 days depending on the species [39]). 2) There is no variation in immunity among birds. 3) Due to the relative helplessness of nestlings, lack of feathers in the first 6-7 days of life [30], and the daily biting habits of black flies (while parents are foraging), nestlings are assumed to be more accessible to biting vectors; thus, we assume that all birds experience the same black fly biting rate, however, the probability of transmission from an infectious vector is higher for nestlings (*b*_*N*_) than for feathered birds (*b*_*F*_) in this model. 4) Increased mortality associated with parasitic infection occurs when birds are acutely infectious. 5) Black fly mortality does not differ based on reproductive stage or stage of infection. 6) Individuals in each module interact through random mixing.

### Solution and analysis of the models

To solve the models we used a fourth-order Runge-Kutta algorithm [44] in Berkeley-Madonna [45], a software package designed to analyze dynamical systems models. The single season model ran across a transmission season of 200 days while the extended model included multiple transmission seasons and years (365 days). To simulate overwintering bird populations in the extended, multi-year model we used four *ovens*, or compartments for overwintering birds of different age (first year and adult birds) and infection statuses (*susceptible* or *infected*, Text S3). *Ovens*, in Berkeley-Madonna, are compartments that fill with individuals for a given amount of time (*fill time*), hold them for a specified time interval (*cook time*), and then release all individuals simultaneously into a compartment (see Text S3 for command syntax). Susceptible and latently infected first year and adult birds each enter into the corresponding overwinter *oven* at the end of the breeding season (day 200).

We conducted two kinds of sensitivity and uncertainty analyses to determine the relative influence of the 23 model parameters on transmission dynamics across a five year span of time [46,47] on two output estimates representative of transmission in this system: the final prevalence of infected birds and the maximum prevalence of infectious black flies in the fifth transmission system. We present both analyses to assess the sensitivity and prediction uncertainty for each parameter. We then use our sensitivity and uncertainty analyses, in concert with our field estimates of avian prevalence, to infer reasonable values for parameters that have large effects on avian and black fly prevalence, are highly uncertain, and are difficult to empirically measure in the field.

#### Sensitivity analyses

Sensitivity analyses quantify how changes in the value of input parameters alter the value of an outcome variable [46-48]. The sensitivity analysis, which is the simplest approach, involved changing the value of each parameter while holding the other parameters fixed. This parameter sensitivity measure (elasticity) was estimated by determining the mean percentage change in these outputs from a 1% change in the parameter of interest. This approach is quick and simple, but it explores only a small region of the full parameter space and requires some confidence in the values of the unchanged variables.

#### Uncertainty analyses

Uncertainty analyses assess the prediction imprecision in the outcome variable due to uncertainty in estimating the value of input parameters [46,47]. To explore the effect of varying the values of all input parameters across their whole range simultaneously, we used Hypercube Sampling (LHS) and partial rank correlation (PRC) [47,48]. In LHS, a probability distribution for each of the *K* input parameters (*K* = 23) is chosen (see Text S4), and then the support of each of these distributions is divided into *N* equivalent segments (*N* = 100). We then ran the model *N* times to compute the outcome(s) of interest, each time choosing at random, without replacement, a value for each of the *K* parameters from the *N* subintervals. After these *N* runs, each of the *N* subintervals comprising the distribution for each of our *K* parameters has been sampled once, and only once.

To carry out the partial rank correlation (PRC) process, we first verified that the output variables varied monotonically with each of the input variables. We then assigned ranks to our *N* subintervals for each of our *K* parameters from 1 to *N* in the natural order. Let *f*_*i*_(*k*) be the rank of the interval chosen in run *i* (*i*=1,..*N*) for input parameter *k* (*k*=1,..,*K*) The *f*_*i*_(*k*)s yield an *N* by *K* integer-valued matrix representing the input choices. Next we constructed an *N* x 1 matrix of the output variables generated by each of these *N* runs and then rank-transformed this column matrix by replacing the value of the outcome variable of the *i* ^*th*^ run by its relative size (rank) among the *N* output variables of all *N* runs. Finally, we used the rank-transformed input matrix and the rank-transformed output matrix to calculate the Spearman or rank correlation coefficient and the partial rank correlation coefficient (PRCC). The magnitude of the PRCC indicates the importance of the uncertainty in predicting the value of the outcome variable, and the result for each input and output variable will be a number between -1 and +1. The sign of the PRCC indicates whether the input parameter has a negative or positive effect on the outcome variable (for details see [46,49]).

## Results

The parameter values we chose for our model yielded a final prevalence of infected birds of 30% and a mean prevalence of infectious black flies of 3%, values that match the empirical data. The sensitivity analysis (elasticity) suggests that 13 of the 23 parameters had only weak effects on any of the avian and black fly prevalence; they resulted in less than a half percent change in the output statistics with a one percent change in the parameter value. These included 10 avian specific parameters and 3 black fly specific parameters. Nine parameters affected both avian and black fly prevalence; the black fly output statistic was on average the most sensitive to changes in the model parameters (Table 2 and Text S5 for associated parameter plots). In terms of PRCCs, only 6 of the 23 parameters were significant to 0.001, and one more parameter was significant to 0.01 (Table 3). Five of these six parameters were significant sources of uncertainty for *both* the avian and black fly prevalence. Only *b*_*A*_, the infectivity of acutely infectious birds to susceptible black flies, and *y*_*A*_, the proportion of latently infected adults that return to relapse the following season, were significant sources of uncertainty for avian and black fly prevalence, respectively (Table 3). We did verify that these output variables were indeed monotone functions of each of the 23 input parameters.

**Table 2:** Ranked Elasticities (absolute values sorted in descending order).

**avian prevalence**					**black fly prevalence**			
*Avian*		*Black Fly*			*Avian*		*Black Fly*	
A_B_	**-1.14**	r	**1.94**		δ	**-3.02**	q_F_	**-2.68**
b_F_	**0.92**	q_F_	**-1.76**		q_B_	**-1.11**	d_4_	**-2.09**
δ_R_	**-0.89**	d_4_	**-1.37**		δ_R_	**0.76**	r	**2.00**
c_B_	**-0.60**	b_R_	**0.79**		λ_B_	**0.65**	c_F_	**-1.62**
*X*	-0.19	c_F_	**-0.66**		γ_A_	**-0.55**	b_R_	**1.28**
γ_A_	0.15	λ_F_	0.35		*X*	-0.26	b_A_	0.31
q_B_	0.13	b_A_	0.09		d_3_	-0.16	λ_F_	0.28
d_1_	0.10	b_C_	0.01		b_F_	0.14	b_C_	0.05
δ	-0.06	A_F_	0.00		γ_J_	0.10	A_F_	0.00
b_N_	0.05				d_2_	0.07		
γ_J_	0.02				A_B_	0.02		
d_3_	-0.01				b_N_	-0.01		
λ_B_	0.01				d_1_	-0.01		
d_2_	0.00				c_B_	0.00		

Avian prevalence = final avian prevalence of infection in the fifth transmission season. Black fly prevalence = peak black fly infection prevalence in the fifth transmission season.

**Table 3:** Uncertainty of model parameters (ranked in descending order).

	**PRCC**	**p-value**
***finalprevalenceofinfectedbirdsinyear5***		
*d*_*4*_ (natural death rate of black flies)	-0.8789	0.001
*b*_*F*_ (infectivity of an infectious black fly to a feathered bird)	0.6912	0.001
*r* (the number of bites a black fly takes per day)	0.5876	0.001
*q*_*F*_ (the date of peak black fly emergence)	-0.5744	0.001
*b*_*R*_ (infectivity of relapsing infectious birds to susceptible black flies)	0.4892	0.001
*b*_*A*_ (infectivity of acutely infectious birds to susceptible black flies)		
***peakprevalenceofinfectiousblackfliesinyear5***		
*d*_*4*_ (natural death rate of black flies)	-0.9066	0.001
*y*_*A*_ (proportion of latently infected adult birds that return to relapse)	0.6784	0.001
*q*_*F*_ (the date of peak black fly emergence)	-0.6096	0.001
*b*_*R*_ (infectivity of relapsing infectious birds to susceptible black flies)	0.4561	0.001
*b*_*F*_ (infectivity of an infectious black fly to a feathered, susceptible bird)	0.4249	0.001
*r* (the number of bites a black fly takes per day)	0.4112	0.001

Parameter elasticities and uncertainties suggest that estimating the natural death rate (*d*_*4*_), or equivalently the average lifespan, of black flies in this system is most critical for precisely predicting prevalence of infection in birds and black flies at the end of five years. The date of peak black fly emergence (*q*_*F*_), the infectity of black flies to feathered birds (*b*_*F*_), and the average number of fly bites per day per female fly (r) also have strong effects on the prediction precision of both avian and black fly prevalence.

### The contact parameter *r*

In disease models, the parameter governing the contact rate between susceptible and infectious individuals can have large effects on transmission dynamics in the system. In these models, both avian and black fly prevalence were sensitive to the parameter *r*, the number of bites a female black fly takes per day. The PRCC analysis indicated that the value of *r* contributes to the uncertainty of our estimates of both output statistics (PRCC = 0.5876, third most important for end of season avian prevalence; PRCC = 0.4112; fifth most important for peak black fly prevalence). In terms of elasticities, the parameter *r* was the most sensitive parameter for avian prevalence and the fourth most sensitive for black fly prevalence. One percent increases in *r* resulted in increases of nearly 2% for both the avian and black fly prevalence. For *r* values greater than one, the end of season avian infection prevalence was no longer sensitive (Table 2, Text S5); however, an *r* of one unreasonably assumes that individual black flies lay eggs every day. Even though an *r* of 0.13 is reasonable for *S. silvestre* */ craigi* (it assumes female black flies experience 4 reproductive cycles throughout their lifespan), we explored how the model performs with other ecologically realistic values for *r*. We ran two different simulations assuming female black flies experience five to six reproductive cycles on this site (*r* = 0.17 and *r* = 0.20). Both of these models yielded unrealistically high final prevalences of infected birds (45%-55%; Text S5). Thus, even though *r* has a high PRCC, the model suggests that in this system an *r* of 0.13 (four reproductive cycles) appears to be the most realistic value.

### The probability of transmission between host and vector

Our model included two transmission parameters from infectious black flies to susceptible birds (*b*_*N*_ and *b*_*F*_) and three transmission parameters from birds of different infectious classes to susceptible black flies (*b*_*A*_, *b*_*C*_, and *b*_*R*_). Two of these parameters (*b*_*N*_ and *b*_*C*_) had very low elasticities and did not have significant PRCCs (Tables 2 and 3), suggesting that transmission from nude nestlings and chronically infectious birds play only a minor role in the transmission dynamics at this site. In contrast, *b*_*F*_ (infectivity of infectious black flies to feathered birds) and *b*_*R*_ (infectivity of relapsing infectious birds to susceptible black flies) had moderately large, positive, significant PRCCs and high elasticities (Tables 2 and 3); this suggests that both avian and black fly prevalence were sensitive to changes in these parameters, whose estimates are subject to high uncertainty. The fifth transmission parameter *b*_*A*_ (infectivity of acutely infectious birds to susceptible black flies) had a PRCC significant to 0.01, but not 0.001 (Table 3), suggesting that this parameter had moderate uncertainty for avian prevalence only. The sensitivity analysis suggests that while *b*_*A*_ is relatively uncertain, it does not strongly affect the dynamics of end of season avian and peak black fly prevalence in the fifth season (low elasticities; Table 2).

### Compartmental transition and death parameters

There are four death rate parameters in our model: *d*_*1*_, the natural death rate of nude nestlings; *d*_*2*_, the natural death rate of feathered birds; *d*_*3*_, the parasite-induced death rate of acutely infectious birds; and *d*_*4*_, the natural death rate of black flies. The death rate parameters for the avian populations all have very low elasticities and non-significant PRCCs suggesting they contribute little to transmission dynamics on these sites. In contrast, *d*_*4*_ has the largest, most significant PRCC and the third largest elasticity for both avian and black fly prevalence. Certainly, the lower the death rate (longer lifespan) for black flies, the higher the probability of a black fly becoming infected and surviving to transmit the infection before death. The highly significant PRCC value associated with this parameter indicates that the accurate estimation of the natural death rate of black flies is crucial for predicting avian and black fly prevalences.

There are a number of parameters that model the rates birds and black flies transition through various stages of infection and life history during the breeding season. None of these parameters were found to have significant PRCCs except for the rate a relapsing infectious bird becomes a latently infected bird (*δ*_*R*_); this parameter had the highest elasticity for black fly prevalence and is also an important driver for parasite prevalence in the bird population. Interestingly, avian and black fly prevalence was also influenced by the proportion of latently infected adults that return to relapse the subsequent season (*y*_*A*_), which displayed high elasticity and PRCC scores (Tables 2 and 3).

### Parameters of the nestling hatch and black fly emergence functions

We used a number of parameters to describe the periods in which nestling birds hatch and black flies emerge each season: *A*_*B*_ and *A*_*F*_, the number of hatching / emerging birds and black flies; *c*_*B*_ and *c*_*F*_, the length of the hatching / emerging periods; and *q*_*B*_ and *q*_*F*_, the peak date of nestling hatching and black fly emergence. Of these parameters, only the date of peak black fly emergence (*q*_*F*_) had a significant PRCC and high elasticity for both avian and black fly prevalence (Tables 2 and 3), suggesting it is a parameter that requires careful estimation. As *q*_*F*_ shifts one percent later in the transmission season away from the date suggested by field estimates (day 66), there was a substantial decrease in the avian and black fly prevalence statistics by 1.7 and 2.7 percent, respectively (Text S5). Black fly emergence duration *c*_*F*_ while displaying a non-significant PRCC, showed a moderate elasticity score (Table 2), the length of the nestling hatch period *c*_*B*_ also displayed only moderate elasticities and non-significant PRCC scores (Tables 2 and 3).

## Discussion

Empirical data collected during the summers of 2005 indicate that the end of the season prevalence of sparrows infected with *L* *. fringillinarum* (comprising acute, relapsing and chronic infections) on these sites is on average 30% (Figure 1). Our model does estimate the final prevalence of infected birds to be 30% for the parameter values chosen. However, if our field sample contains some chronically infectious birds across each summer, the empirical data may underestimate prevalence, and our model estimate may be too low; our visual scoring method can miss infections with low parasitemia (1-3 gametocytes per 10,000 red blood cells), resulting in false negatives. Thus, true prevalence of infected sparrows on these sites may indeed be higher within a transmission season.

The model estimate of approximately 5% for mean prevalence of infectious black flies within a season is reasonable, even though there is a considerable lack of empirical data on the prevalence of *Leucocytozoon* spp. in black fly populations. In Algonquin, Ontario, 90% to 100% of all ornithophilic black fly species had *Leucocytozoon* sporozoites present in their salivary glands [50]. Hellgren et al. [51] found 62% of blood-fed black flies (n = 38) of different species were positive for *Leucocytozoon* spp. infections. However, once black flies and *Leucocytozoon* parasites are separated into different species, the prevalence of infectious black flies infected with a particular species of *Leucocytozoon* is certainly much lower. Furthermore, the prevalence of infectious mosquitoes in areas of high endemicity for human malaria is surprisingly low, with prevalences of 2.7% and 2.1% for *P. falciparum* and *P. vivax*, respectively [52].

### Estimation of parameters influencing parasite prevalence

The sensitivity and uncertainty analyses on all 23 model parameters indicated that the end of season prevalence of infected birds and the peak black fly prevalence in the fifth transmission season were both significantly sensitive to eight parameters, five of which require careful estimation with empirical data due to high levels of uncertainty (Table 3). Assuming our estimates for the other model parameters are reasonable, we can use model performance to infer estimates for parameters of high uncertainty in our model that have small regions of values that generate realistic estimates for end of season avian prevalence (30%).

For example, a value of 0.213 for *r* (the number of bites a female black fly takes per day) yielded an average end of season avian prevalence of 30%. Values of *r* that were less than or greater than 0.213 yielded unreasonably low and high infection prevalences, respectively, in the sparrow population (Text S5). Assuming that black flies take one bite per gonotrophic cycle, these results suggest that four-five gonotropic cycles across a maximum 30 day lifespan is potentially the most realistic value for this system. Both avian and black fly prevalences were highly sensitive to the natural death rate of black flies (*d*_*4*_); like *r*, this parameter estimate had high uncertainty and is very difficult to measure in the field. In the sensitivity analysis, values of *d*_*4*_ that range from 0.032 to 0.107 dying black flies per day (average lifespans of 9.35-31 days) generate avian end of season prevalences of 25-35%. This suggests that our current value in the model for *d*_*4*_ (0.17 flies dying per day or an average lifespan of approximately 6 days) is probably a bit too high, and a value of 0.066 flies per day (15 days) may be more realistic, generating an average end of season avian prevalence of 30%. The model estimate for *q*_*F*_ (the peak date in black fly emergence) is probably a realistic one, regardless of high uncertainty associated with this parameter, because we used field capture data specifically for *S. Silvestre / craigi* from these sites to generate this estimate. In contrast, the probabilities of transmission from host to vector (*b*_*A*_) and from vector to host (*b*_*N*_ and *b*_*F*_) are important parameters in every transmission model, but are especially difficult to estimate in the field and have many values that generate somewhat reasonable estimates of avian prevalence at the end of the season.

### The role of parasite relapse and first year birds for parasite persistence

Both the relapse of previously infected birds and susceptibility of first year birds are crucial in maintaining seasonal transmission on the breeding grounds. Changes in the parameters associated with relapsing infectious birds (*b*_*R*_, *δ*_*R*_, and *y*_*A*_) significantly influenced both the end of season prevalence of infected birds and the peak prevalence of infectious black flies in the fifth transmission season. More precisely, increases in the proportion of returning adults that relapse (*y*_*A*_) and the rates of transmission from relapsing infectious birds to susceptible black flies (*b*_*R*_) significantly increased both avian and black fly prevalence. In contrast, increases in the rates relapsing infectious birds become latently infected (no longer infectious, *δ*_*R*_) significantly decrease avian and black fly prevalence. This is not surprising, considering that latently infected birds that return to breed and relapse represent a significant reservoir of parasites to emerging black flies. Thus, the lower the proportion of returning, relapsing infectious adults, and the faster these adults are removed from the infectious class, the less infection will spread to and be transmitted from the black fly population to susceptible first year birds. In this model we assumed that all latently infected overwintering birds returned to the breeding grounds and relapsed (*y*_*A*_ = 1). However, this assumption may not be entirely valid, and the probability of relapse may depend on the relationship between body condition, breeding demands, and seasonal changes in corticosterone [53-57]. Due to the high uncertainty associated with this parameter, estimating the proportion of returning infected adults that relapse prior to the emergence of biting vectors is probably important for understanding seasonal transmission dynamics.

In contrast, infection prevalences in both populations were insensitive to changes in parameters associated with chronically infectious birds (*b*_*C*_). This may be due to the fact that the peak of the black fly emergence curve (the peak of susceptible black flies) does not tightly overlap with the peak of chronically infectious first year birds, which occurs later in the season. In each season, the majority of chronic infections are due to the susceptible first year birds becoming infected for the first time. Thus, chronically infectious birds may play only a minor role in transmission dynamics at this site for a couple of reasons: 1) chronic infections are associated with low parasitemia and transmissibility [29], and 2) the timing of the emergence and peak of susceptible vectors occurs before birds begin to develop chronic infections.

**Figure 3:**
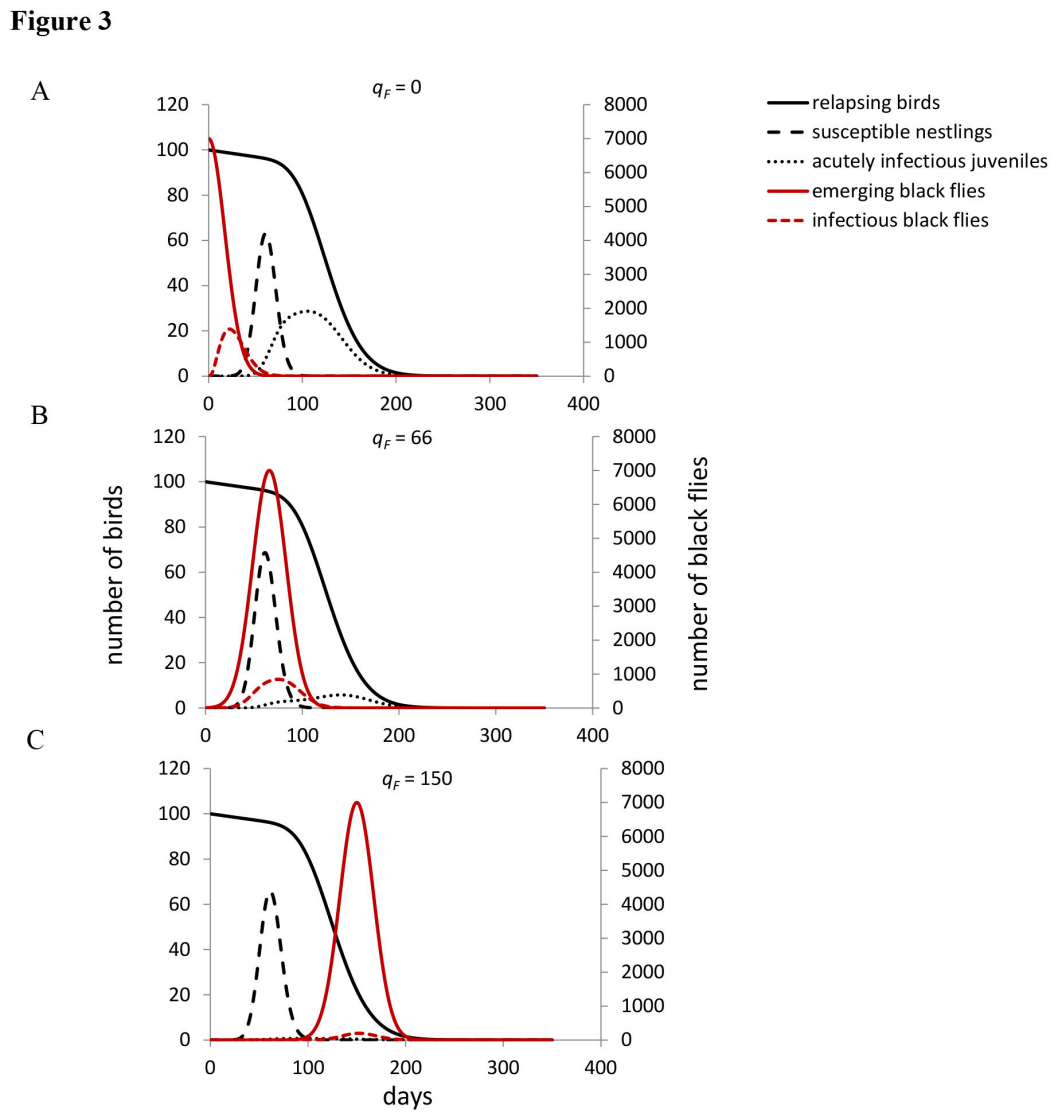
Earlier black fly emergence increases parasite prevalence in sparrow and vector populations. As suggested by empirical data, *q*_*F*_ was initially set at day 66 (July 5th). We then shifted *q*_*F*_ earlier (day 0, May 1st) and later (day 120, August 28^th^) in the season. Both final prevalence of infected birds and mean prevalence of infectious black flies was highest when *q*_*F*_ was set to day 0 because this maximized the degree of overlap between susceptible first year birds and infectious black flies.

The extended model suggests that first year birds play an important role in persistence of seasonal transmission of *L* *. fringillinarum* . The degree of seasonal overlap between susceptible first year birds and infectious black flies significantly affected both the final prevalence of infected birds and the mean prevalence of infectious black flies (Figure 3). First year birds, especially in temperate systems, represent a pulse of susceptible hosts each season; other analytical studies have demonstrated their significance in maintaining seasonal transmission [58,59]. Adults, once infected, remain infected throughout the remainder of their life span [1,14]. In addition, adults are seasonally faithful to their breeding territories and do not disperse to new areas [36,37]. Thus, new susceptible birds are introduced solely through the nestlings produced each season, or through susceptible adults that had earlier escaped infection. When the possibility of transmission between infectious black flies and first year birds is removed from the model, *L* *. fringillinarum* quickly fades after a couple of seasons due to the exhaustion of susceptible birds.

However, distinguishing between nude nestlings and feathered first year birds in the transmission system is unnecessary, as shown by the insensitivity of both avian and black fly prevalence to the daily rate nestlings acquire feathers (*X*) and the probability of transmission from an infectious black fly to a nude nestling (*b*_*N*_). Nestlings are without feathers for a relatively brief window of time during the breeding season, and an increase in the probability of transmission during this interval may not significantly affect overall infection in both the bird and black fly population. This contrasts with results from models for St. Louis encephalitis virus [58,60], West Nile virus [60], and Equine encephalitis models [59], which found nestlings to be important for transmission dynamics. However, these models incorporated mosquito feeding preferences for nude nestlings and assumptions incorporating the development of nestling immunity; in the encephalitis systems, nestling birds develop infections faster, maintain higher and longer viremias than adult birds, and consequently are good amplifying hosts in these systems. Future models of this system could incorporate these assumptions.

### Potential effects of environmental variation on prevalence in high elevation systems

Some of the most influential parameters in this model affecting parasite prevalence in both the sparrow and black fly population were associated with the black fly vector. A multitude of studies have demonstrated that dipteran development is sensitive to changes in ambient temperature, with temperature increases within some range resulting in an increase in dipteran growth, development, metabolic rate, and gonotrophic cycles [8,39,61-63]. Parasite development in the vector also has been shown to increase with temperature within some range [1,64,65]. While we did not model this explicitly (by incorporating temperature-dependence into model parameters), we can begin to make some initial predictions for future models on how seasonal variation may influence parasite prevalence through potential temperature-mediated shifts in black fly emergence and biting rates.

For example, the timing of black fly emergence has been shown to vary with overwinter snowfall; black fly emergence can shift up to a month later due rivers and creeks being covered with snow for longer durations following winters with high snowfall (effectively insulating river temperatures from warming air temperatures [66]). In contrast, avian timing of reproduction is minimally influenced by changes in temperature; reproduction is regulated instead by seasonal elevations in corticosterone and sex hormones, which are driven by increasing day length [67-69]. Thus, in particularly warm springs due to low snow fall overwinter, black flies may emerge earlier. Under this scenario, our model suggests that warmer seasons could in fact increase parasite prevalence because shifting the date of peak black fly emergence (*q*_*F*_) earlier in the season, to a point, significantly increased the degree of overlap between infectious black flies (infected from feeding on relapsing infectious adult sparrows) and susceptible first year birds (Figure 3). Shifting the date later in the season minimized this overlap and resulted in fewer acute infections in the first year bird population. Thus, the timing of peak black fly emergence, parasite relapse, and when nestlings are produced should have implications for parasite prevalence and persistence across seasons. These effects might be even more dramatic when combined with any temperature-mediated changes in black fly gonotrophic cycles / biting rates (*r*, Text S5), as has been shown in mosquito-malaria systems [8,70].

Interestingly, in contrast to empirical data for human malaria and bird malaria in the Hawaiian archipelago (e.g. *P. falciparum* [71-73]; *P* *. relictum* [74]), temperature-mediated increases in parasite development within the black fly vector (*λ*_*F*_) may not necessarily increase parasite prevalence (Table 2 and 3) in this system. Parasite prevalence in both the bird and black fly population was insensitive to changes in this parameter. This is mostly likely because the extrinsic incubation period for *Plasmodium* parasites is much longer with respect to the lifespan of the mosquito vector with many mosquitoes dying before they can transmit the parasite. In contrast, the parasite development rate of *Leucocytozoon* spp. is much faster and could comprise a smaller portion of the black fly vector’s lifespan. Therefore, most black flies may survive to transmit *Leucocytozoon* parasites in the field, and temperature-mediated increases in parasite development rate within the vector may not significantly increase parasite transmission overall.

### Implications and future directions

With this multi-year model, we present an in depth look at the ecological, parasite, and host factors that are important for parasite transmission outside of the well-described Hawaiian Island bird malaria system. We conclude that seasonal relapse is crucial for parasite transmission across years. Further, parasite prevalence in the bird population is highly sensitive to the timing of black fly emergence, seasonal bird relapse, and the production of susceptible first year birds. Thus, changes in environmental temperature due to inter-seasonal variation in overwinter snowpack and black fly ecology could be critical determinants of parasite prevalence and sparrow recruitment from year to year.

Further research and better precision of model parameters are needed to improve some of our model predictions. White-crowned sparrows and *L* *. fringillinarum* live in a system comprised of multiple vertebrate hosts, potentially multiple black fly vectors, and other blood parasite species. For example, we have found a range of other bird species infected with the same morphospecies of *L* *. fringillinarum* , as well as the presence of other co-infecting blood parasite species (e.g. *Plasmodium* , *Parahaemoproteus,* and *Trypanosoma* spp) on these sites [38]. How multiple bird hosts, black fly vectors, and potentially competing parasites interact in space and time could profoundly influence transmission dynamics in this system. Further, we know very little about how black fly biting rates may vary among birds of different ages [13], sex [13], stage [75,76], and species or on non-avian hosts [51,77,78]. Finally, it would also be interesting to more rigorously include relevant environmental factors, such as interactions between climate change and elevation (which have been shown to have strong effects on the transmission ecology of temperate and Hawaiian bird malaria [8,74]), variation in seasonal temperature and snowpack data, as well as bird proximity to black fly larval habitat [13,79].

## Supporting Information

Text S1 **Description of field methods for capturing birds, black flies, and sampling parasite prevalence throughout each breeding season.** Details on parameterizing the nestling hatch and black fly emergence function are also included.
(DOC)

Text S2 **Compartmental diagram and differential equations associated with the single season model.**
(DOC)

Text S3 **Differential equations for the multi-season model.**
(DOC)

Text S4 **Includes a table of the distributions assumed for each model parameter as well as detailed descriptions on choice of parameter distributions, minimum, maximum, and mean values.**
(DOC)

Text S5 **Parameter plots of the parameters avian and black fly infection prevalence were most sensitive to as indicated in the sensitivity analysis.**
(DOC)
