# Supplementary material for: A Transmission Model for the Ecology of an Avian Blood Parasite in a Temperate Ecosystem
Source: PLoS One. 2013 Sep 20;8(9):e76126. doi: 10.1371/journal.pone.0076126 (PMC3779181; doi:10.1371/journal.pone.0076126)
Supplement: Text S1 — Description of field methods for capturing birds, black flies, and sampling parasite prevalence throughout each breeding season. Details on parameterizing the nestling hatch and black fly emergence function are also included. (DOC) [file pone.0076126.s001.doc]

**Text S1**

**Empirical Collection Methods and Parameterization of Nestling and Black Fly Emergence Functions**

*Field methods for collection of empirical data relevant to model construction*

Two field sites were located approximately two km south (UTM: N 4312713 E 327700) and 2 km north from RMBL (UTM: N 4314126.9 E 327648.3), and the third field site was located in the adjacent, Washington Gulch valley (UTM: N 4311531 E 325807). Areas of each field site ranged from 12.3 – 19.7 ha. Elevation of the three study sites ranges between 2902 m to 2987 m above sea level. During the summers of 1999 – 2008, we captured sparrows between 06:00 and 12:00 each day in millet-baited, single- and double-cell (18 cm x 18 cm x 18 cm), galvanized wire Potter traps. Sparrows were marked with a unique combination of three colored leg-bands and one metal U.S. Fish and Wildlife Service band (Bird Banding Laboratory, Patuxent, MD, USA). Upon capture, birds were sexed, standard morphometric measures were collected, and blood was sampled from the brachial vein with a sterile 26-gauge hypodermic needle (Fisher Scientific Precision Glide, No. 14-826-15, Pittsburgh, PA, USA) to determine infection status. We collected blood samples in 70 μL heparinized microcapillary tubes (Fisher Scientific, No. 22-362-566, Pittsburgh, PA, USA) and temporarily stored them in a cooler on ice. A small portion of the blood sampled from each bird was placed on a microscope slide (Fisher Scientific, No. 12-542-5, Pittsburgh, PA, USA), air-dried, and stained with a Fisher Hema 3 Stat pack^TM^ (Fisher Scientific, No. 22-122911, Pittsburgh, PA, USA). *Leucocytozoon* parasites were identified to species [1] and we counted the total number of gametocytes out of 10,000 red blood cells to quantify intensity of infection. To estimate monthly and seasonal field prevalences of infection in the bird population, we divided the number of birds positive for *Leucocytozoon*  *fringillinarum* parasites by the total number of birds captured across each month and in each field season.

Each year we searched for nests from June 5 through July 30. Nests were located during building, egg-laying, incubation, and feeding of nestlings via territory searches and bird behavioral cues [2]. Plots were searched every three days to eliminate temporal bias. For each nest, the color-band combinations, if available, of the associated male and female, were recorded. Nests were checked once every two to three days and date, time, stage of nest, number of eggs or young and estimated age of the young were recorded. Seven days after hatching, nestling mass and tarsus was measured [3] because nestlings usually fledge on day nine. We categorized nests as “successful” if one or more of the following pieces of evidence was observed: a flattened nest rim and fecal droppings in the nest, nearby adult alarm calls, begging calls of the young, or observed fledgling feedings [2]. For failed nests, “failure” was recorded as “predation” if there were signs of predator disturbance and missing eggs or nestlings or “desertion” if cold eggs or dead nestlings remained in the nest.

To ensure a representative sample of the black fly community in our field sites, we sampled black flies across four, eight day intervals from May 15^th^ – July 31^st^ in 2005 and 2007, across a variety of common habitats. Each field site was first stratified by the following broad habitat types: willow, alpine meadow, and forest habitat patches. We then selected a random sample of the habitat patches from each field site. Black flies were collected in carbon-dioxide baited Center for Disease Control (CDC) miniature light traps (John W. Hock Company, No. 512 fine mesh collection cups, Gainesville, FL, USA) [4], which were set at 9:00 AM and checked at 9:00 AM every 24 hours. Due to the chance of trap failure, traps were paired approximately 50 m from each other within each sampled patch. Each habitat patch was trapped for two consecutive nights over an interval of eight days. After each two-day trapping session, we pulled traps and rotated them to the other field site to minimize any potential effects of season or weather on trap success. Within two hours of capture in the field, all biting dipterans were placed into a garbage bag and exposed to cotton soaked in triethylamine (Fisher Scientific, Amber Glass, No. BP616-500, Pittsburgh, PA, USA) for five minutes in a well-ventilated area. Once flies were immobilized, black flies were separated out from other biting dipterans and stored immediately in 95% ethanol for future identification and parasite DNA analyses. Female black flies were identified to species or species complex based on structural characters presented in the keys and illustrations of Adler et al. [5]. Identifications were facilitated by genitalic preparations of selected specimens.  Representative specimens have been deposited in the Clemson University Arthropod Collection, Clemson, South Carolina.

*Parameters associated with nestling hatch and black fly emergence functions*

The parameters associated with the time dependent nestling hatch and black fly emergence functions were estimated from empirical data collected from the White-crowned Sparrow and *S. silvestre / craigi* populations living on our field sites. Nest monitoring data were compiled from 2003 and 2004 to determine the number of nestlings hatched per female per day. This generated a curve with the peak number of nestlings hatching female^-1^ day^-1^ falling approximately on day 57 (June 26^th^) of each breeding season. We used the following exponential / quadratic function to approximate the curve generated from the empirical data,

$\frac{B_{A}A_{B}}{2\left(e^{\frac{\left(t-q_{B}\right)^{2}}{c_{B}}}\right)}$ *A.1*

The above expression is multiplied by the total number of breeding adults (*B_A_*) divided in half to represent the number of nestlings that hatch per female per day. We determined the height of the nestling hatch curve, the peak number of nestlings that hatch per female per day (*A_B_*), by taking the maximum number of nestlings hatching daily (nine per day) and dividing by the total number of breeding females (54). We estimated the parameters for the spread of the nestling hatch function (*c_B_*) and the day the peak number of nestlings that hatch per female per day (*q_B_*) by matching the spread and peak day of the model generated curve to the spread and peak day of the empirical data curve (Figure 1).

We determined an empirical curve from the number of *S. silvestre / craigi* captured in CDC light traps on a daily basis from summers of 2005 and 2007. We then fit an exponential / quadratic curve to control how and when black flies emerge throughout the transmission season in this model.

$\frac{A_{F}}{\left(\frac{\left(t-q_{F}\right)^{2}}{e^{c_{F}}}\right)}$ *A.2*

The height of the black fly emergence function (*A_F_*), the spread of the emergence function (*c_F_*), and the day when peak number of black flies emerge (*q_F_*) were determined by matching the model curve to the empirical curve for *S. silvestre / craigi* daily abundance (Figure B). The exponential curve at first does not appear to fit the empirical trapping data very well; however, after further investigation troughs in the empirical data were determined to really coincide with decreased trapping effort.

**Figure 1 Distributions for black fly emergence and nestling hatching throughout the breeding season.** The daily nestling **(A)** and black fly field data **(B)** overlapping with the corresponding nestling hatch and black fly emergence functions are shown below. Empirical field data used to parameterize both functions are represented by gray bars, while the nestling hatch and black fly emergence functions are represented by black bars. Day zero corresponds to May 1^st^ in both figures. Troughs in the empirical black fly capture data correspond to intervals of decreased trapping effort.

**A**

**B**

**Additional avian hosts and black fly vectors present on these sites**

**Table 1** is a summary of the different black fly species captured in carbon-dioxide CDC baited light traps and their abundances across two field sites [6].

| **Black fly species** | **East River** |  | **Washington Gulch** |
| --- | --- | --- | --- |
| ***Greniera denaria*** | 29 |  | 30 |
|  |  |  |  |
| ***Helodon onychodactylus* cpx** | 5 |  | 3 |
|  |  |  |  |
| ***Metacnephia jeanae*** | 70 |  | 47 |
|  |  |  |  |
| *Prosimulium exigens* | 194 |  | 65 |
| *Prosimulium fulvum* | 1 |  | 0 |
| *Prosimulium hirtipes* group | 22 |  | 43 |
| *Prosimulium uinta* | 0 |  | 1 |
|  |  |  |  |
| *Simulium arcticum* cpx | 339 |  | 175 |
| ***Simulium canonicolum*** | 91 |  | 31 |
| *Simulium decorum* | 0 |  | 2 |
| ***Simulium exulatum / pilosum*** | 112 |  | 97 |
| *Simulium hunteri* | 0 |  | 1 |
| *Simulium irritatum / venustum* | 25 |  | 34 |
| *Simulium piperi* | 19 |  | 20 |
| ***Simulium silvestre / craigi*** | 861 |  | 553 |
| *Simulium vandalicum* | 19 |  | 19 |
| *Simulium vittatum* cpx | 6 |  | 7 |
|  |  |  |  |

**Table 2** is a summary of the different bird species and their abundances present on these two field sites. Data were generated from point counts across these two field sites. Bird species highlighted in bold have had bloodsmears positive for the morphospecies *Leucocytozoon fringillinarum* [6].

| **Family** | **Species** | **RMBL Counts** |  | **WA Counts** |
| --- | --- | --- | --- | --- |
| Accipitridae | Cooper's Hawk | 1 |  | 0 |
|  | Red-tailed Hawk | 0 |  | 1 |
|  | Sharp-shinned Hawk | 0 |  | 3 |
|  |  |  |  |  |
| Cardinalidae | Black-headed Grosbeak | 1 |  | 6 |
|  | Lazuli Bunting | 25 |  | 3 |
|  |  |  |  |  |
| Charadriidae | Killdeer | 0 |  | 5 |
|  |  |  |  |  |
| Columbidae | Mourning Dove | 0 |  | 5 |
|  |  |  |  |  |
| Corvidae | Common Raven | 4 |  | 2 |
|  | Gray Jay | 0 |  | 3 |
|  |  |  |  |  |
| **Emberizidae** | Chipping Sparrow | 1 |  | 2 |
|  | **Fox Sparrow** | **41** |  | **12** |
|  | Gray-headed Junco | 11 |  | 26 |
|  | **Green-tailed Towhee** | **15** |  | **1** |
|  | **Lincoln's Sparrow** | **40** |  | **64** |
|  | **Mountain White-crowned Sparrow** | **36** |  | **70** |
|  | Savannah Sparrow | 0 |  | 10 |
|  | Song Sparrow | 7 |  | 0 |
|  | **Vesper Sparrow** | **3** |  | **4** |
|  |  |  |  |  |
| Fringillidae | Cassin's Finch | 0 |  | 1 |
|  | Pine Grosbeak | 1 |  | 3 |
|  | Pine Siskin | 34 |  | 29 |
|  |  |  |  |  |
| **Hirundinidae** | Cliff Swallow | 1 |  | 1 |
|  | **Tree Swallow** | **7** |  | **14** |
|  | **Violet-green Swallow** | **12** |  | **12** |
|  |  |  |  |  |
| Icteridae | Brown-headed Cowbird | 11 |  | 8 |
|  | Red-winged Blackbird | 4 |  | 11 |
|  |  |  |  |  |
| Paridae | Black-capped Chickadee | 1 |  | 0 |
|  | Mountain Chickadee | 8 |  | 18 |
|  | Red-breasted Nuthatch | 0 |  | 4 |
|  |  |  |  |  |
| **Parulidae** | Audubon's Warbler | 16 |  | 20 |
|  | **MacGillivray's Warbler** | **29** |  | **7** |
|  | Wilson's Warbler | 18 |  | 11 |
|  | **Yellow Warbler** | **56** |  | **41** |
|  |  |  |  |  |
| Picidae | Downy Woodpecker | 2 |  | 1 |
|  | Hairy Woodpecker | 1 |  | 12 |
|  | Red-naped Sapsucker | 15 |  | 25 |
|  | Red-shafted Flicker | 18 |  | 15 |
|  |  |  |  |  |
| Regulidae | Golden-crowned Kinglet | 2 |  | 2 |
|  | Ruby-crowned Kinglet | 9 |  | 24 |
|  |  |  |  |  |
| Scolopacidae | Common Snipe | 0 |  | 3 |
|  |  |  |  |  |
| Strigidae | Flammulated Owl | 0 |  | 1 |
|  |  |  |  |  |
| Tetraonidae | Blue Grouse | 4 |  | 7 |
|  |  |  |  |  |
| Thraupidae | Western Tanager | 0 |  | 5 |
|  |  |  |  |  |
| Trochilidae | Broad-tailed Hummingbird | 25 |  | 19 |
|  |  |  |  |  |
| Troglotydidae | House Wren | 34 |  | 19 |
|  |  |  |  |  |
| Turdidae | American Robin | 82 |  | 45 |
|  | Hermit Thrush | 1 |  | 0 |
|  | Mountain Bluebird | 1 |  | 1 |
|  | Swainson's Thrush | 24 |  | 0 |
|  | Townsend's Solitaire | 1 |  | 0 |
|  |  |  |  |  |
| Tyrannidae | Cordilleran Flycatcher | 3 |  | 3 |
|  | Dusky Flycatcher | 13 |  | 7 |
|  | Hammond's Flycatcher | 1 |  | 0 |
|  | Olive-sided Flycatcher | 2 |  | 5 |
|  | Western Wood-pewee | 25 |  | 27 |
|  | Willow Flycatcher | 10 |  | 0 |
|  |  |  |  |  |
| Vireonidae | Warbling Vireo | 44 |  | 52 |
|  |  |  |  |  |

**Associated references:**

[1] Valkiunas, G (2005) Avian malaria parasites and other haemosporidia. New York: CRC Press, p.

[2] Martin, TE, Geupel, GR (1993) Nest-Monitoring Plots - Methods for Locating Nests and Monitoring Success. J of Field Ornithol. 64: 507-519.

[3] Morton, ML (2002) The Mountain White-crowned Sparrow: migration and reproduction at high altitude. Camarillo: Cooper Ornithological Society, 236 p.

[4] Service, MW (1976) Mosquito ecology: field sampling methods. New York, USA: Wiley, 583 p.

[5] Adler, PH, Currie, DC, Wood, DM (2004) The Black Flies (Simuliidae) of North America. Ithaca: Cornell University Press, 937 p.

[6] Murdock, CC (2009) Studies on the ecology of avian malaria in an alpine ecosystem. Ph.D. (University of Michigan, Ann Arbor).
