# Supplementary material for: A Transmission Model for the Ecology of an Avian Blood Parasite in a Temperate Ecosystem
Source: PLoS One. 2013 Sep 20;8(9):e76126. doi: 10.1371/journal.pone.0076126 (PMC3779181; doi:10.1371/journal.pone.0076126)
Supplement: Text S2 — Compartmental diagram and differential equations associated with the single season model. (DOC) [file pone.0076126.s002.doc]

**Text S2**

**Figure 1 The single season model.** The basic model consists of three modules are representing first year birds, adult birds, and black fly vectors. Arrows denote flows of individuals entering or leaving compartments within each module over time. As shown in the module headings, ***B*** and ***F*** represent the total number of individuals in the bird and black fly vector populations, respectively. The first one or two letters of each compartment label corresponds to the infection status of individuals entering or leaving that compartment (***S*** = susceptible, ***E*** = exposed, ***I*** = acutely infectious, and ***CI*** = chronically infectious). The subscripts of compartment label corresponds to the population the module represents (***N*** = nude nestling and ***J*** = feathered first year birds, ***A*** = feathered adult bird population, and ***F*** = black fly vector population). Initial conditions for the compartments in the modules are the following: 300 sparrows begin as susceptible adults (***S_A_***) and 100 sparrows enter as chronically infectious adults (C***I_A_***). All other compartments initially begin with zero individuals. Because susceptible black fly vectors may acquire an infectious bloodmeal from acutely infectious and chronically infectious birds with different transmission potentials, ***P*** is equal to the sum of [*b_A_* (*I_A_* + *I_J_*) + *b_C_* (*CI_A_* + *CI_J_*)] / *B*.

**Single Season Model Equations**

1. Young of the year (YOY) birds: nestlings (*N*) and feathered juveniles (*J*)

*B.1.1* *susceptible nude nestlings*

*B.1.2 susceptible juveniles*

*B.1.3 exposed juveniles*

*B.1.4 acutely infectious juveniles*

*B.1.5 chronically infectious juveniles*

1. Adult birds (*A*)

*B.2.1 susceptible adult birds*

*B.2.2 exposed adult birds*

*B.2.3 acutely infectious adult birds*

*B.2.4 chronic infectious adult birds*

1. Black flies (*F*)

*B.3.1 susceptible black flies*

*B.3.2 exposed black flies*

*B.3.3 infectious black flies*
