# Supplementary material for: A Transmission Model for the Ecology of an Avian Blood Parasite in a Temperate Ecosystem
Source: PLoS One. 2013 Sep 20;8(9):e76126. doi: 10.1371/journal.pone.0076126 (PMC3779181; doi:10.1371/journal.pone.0076126)
Supplement: Text S3 — Differential equations for the multi-season model. (DOC) [file pone.0076126.s003.doc]

**Text S3**

*Differential Equations for the Extended, Multiple Season Model of the Bird (B) and Black Fly (F) populations*

1. First year birds: nestlings (*N*) and feathered juveniles (*J*)

*susceptible nude nestlings*

*susceptible juveniles*

*exposed juveniles*

*acutely infectious juveniles*

*chronically infectious juveniles*

1. Adult birds (*A*)

*susceptible adult birds*

*exposed adult birds*

*acutely infectious adult birds*

*chronic infectious adult birds*

*relapsing infectious adult birds*

1. Black flies (*F*)

*susceptible black flies*

*exposed black flies*

*infectious black flies*

1. Ovens and overwintering bird populations

*susceptible YOY bird overwintering oven*

$\begin{matrix} S_{Jinput}=S_{J15}\gamma_{J} \\ S_{Joven}=oven\left(S_{Jinput},1,359\right) \\ S_{Joutput}=outflow\left(S_{Joven}\right) \\ \frac{dS_{J}}{dt}=S_{Jinput}-S_{Joutput} \end{matrix}$

*susceptible adult bird overwintering oven*

$\begin{matrix} S_{Ainput}=S_{A25}\gamma_{A} \\ S_{Aoven}=oven\left(S_{Ainput},1,359\right) \\ S_{Aoutput}=outflow\left(S_{Aoven}\right) \\ \frac{dS_{A}}{dt}=S_{Ainput}-S_{Aoutput} \end{matrix}$

*latently infected YOY bird overwintering oven*

$\begin{matrix} LI_{Jinput}=CI_{J4}\sigma_{J} \\ LI_{Joven}=oven\left(LI_{Jinput},1,359\right) \\ LI_{Joutput}=outflow\left(LI_{Joven}\right) \\ \frac{dLI_{J}}{dt}=LI_{Jinput}-LI_{Joutput} \end{matrix}$

*latently infected adult bird oven*

$\begin{matrix} LI_{Ainput}=CI_{A4}\sigma_{A} \\ LI_{Aoven}=oven\left(LI_{Aoutput},1,359\right) \\ LI_{Aoutput}=outflow\left(LI_{Aoven}\right) \\ \frac{dLI_{A}}{dt}=LI_{Ainput}-LI_{Aoutput} \end{matrix}$
