# Supplementary material for: A Transmission Model for the Ecology of an Avian Blood Parasite in a Temperate Ecosystem
Source: PLoS One. 2013 Sep 20;8(9):e76126. doi: 10.1371/journal.pone.0076126 (PMC3779181; doi:10.1371/journal.pone.0076126)
Supplement: Text S4 — Includes a table of the distributions assumed for each model parameter as well as detailed descriptions on choice of parameter distributions, minimum, maximum, and mean values. (DOC) [file pone.0076126.s004.doc]

**Text S4**

**Table 1 Parameter distributions**

**Parameter Selection Methods**

***Transmission parameters***

For the probabilities of transmission from an infectious black fly to a susceptible nude nestling (*b_N_*) or feathered bird (*b_F_*), we chose an average value of 0.9 and 0.3, respectively. We estimated *b_F_* from field data on unrestrained White Pekin Ducks (*Anas platyrhynchos*) naturally infected with *Leucocytozoon simondi* from two different sites in northern Michigan . Because we assume in the model that nude altricial nestlings, if encountered by an infectious biting vector, will be less defensive overall and potentially more susceptible than feathered first year and adult birds, we set *b_N_* to a higher value of 0.9, which is similar to transmission probabilities associated with infectious mosquitoes feeding on anesthetized, susceptible birds . We chose a uniform distribution for both parameters with a possible range of 0-1. It is currently unclear whether or not the entire population of overwintering latently infected birds return to the breeding grounds as relapsing infectious birds. Thus, we assumed a uniform distribution for these parameters with a possible range of values of 0-1 birds returning to relapse, and set the average proportion of latently infected first year (*y_J_*) and adult birds (*y_A_*) that return the next season as relapsing infectious adults equal to 0.5.

Assuming that a black fly only takes one bite per gonotrophic cycle, we parameterized the number of black fly bites a bird receives per day (*r*) off of the number of gonotrophic cycles a black fly typically experiences across her lifespan. For similarly related species, this could range from 2-8 cycles across a 30 day lifespan . Because many black flies will not survive to their eighth gonotrophic cycle, we assumed a triangular distribution with a mean value of 0.133 bites per female per day (4 gonotrophic cycles). For the probabilities of transmission from an acutely (*b_A_*), relapsing (*b_R_*), and chronically infectious (*b_C_*) bird to a susceptible black fly, we assumed a uniform distribution ranging with possible values ranging from 0-1. We set an average value of 0.5 for *b_A_* based on infection rates of black flies (*Simulium rugglesi*) fed on 19 naturally infected ducks (*A. boschas*) . We do not have good data on the other transmission probabilities (*b_R_* and *b_C_*), however, relapsing and chronic infections are typically characterized by intermediate and low gametocytemias (3 gametocytes / 10,000 red blood cells) , respectively, and infectivity to the biting vector has been shown to correlate with gametocytemia . Thus, we assumed an average value for *b_R_* or 0.3 and of 0.1 for *b_C_*.

***Avian compartmental transition parameters***

We assumed that the rate an exposed bird transitions into the infectious state (*λ_B_*) was approximately 0.181 birds / day because the average prepatent period lasts approximately 5-6 days , with a minimum and maximum of 4-7 days (0.142 – 0.250 birds / day). We also assumed that this rate was the same for both first year and adult birds and had a triangular distribution. The rate an acutely infectious bird becomes chronically infectious (*δ*) can range from 0.080-0.118 birds / day (acute *Leucocytozoon* infections can range from 8-13 days) . The rate a relapsing infectious bird transitions into the latently infected, overwintering state (*δ_R_*) is relatively slow, because relapsing infections can persist for many months . Due to the compressed breeding season (4 months) in high elevation sites, we assumed that relapsing infections persisted throughout the breeding season until birds migrate for the wintering grounds. Thus, the rate relapsing birds move through each relapsing chain to eventually become latently infected overwintering birds could range from 0.142-0.168 birds per day (divided by 20 chains; 0.0071-0.0084 birds per day; relapsing period = 119-140 days). For both *δ* and *δ_R_* we assumed a normal distribution with a mean model value of 0.095 birds per day and 0.153 birds per day, respectively. Nude nestlings gain down feathers after approximately 6 days post-hatching (0.167 birds per day, ), so we assumed a triangular distribution for this rate parameter with a range of 0.142-0.250 birds per day (1/4 – 1/7 days).

We had some transition parameters that were model programmed (*γ_J_*, *γ_A_*, *σ_J_*, and *σ_A_*) or arbitrarily set to ensure birds of particular states entered the overwintering period at the appropriate time of season. Consequently, we did not include these parameters in our sensitivity / uncertainty analyses. For example, the rate susceptible first year birds transition into the overwinter compartment for susceptible birds (*γ_J_* ) depends on the amount of time that elapses after they hatch. Thus, if the breeding season is 135 days (approximately 4 months), then the time a first year bird is present on the breeding grounds is equivalent to the difference between the length of the breeding season (135 days) and the peak of the nestling hatch function (*q_B_* = day 57). However, the susceptible feathered first year bird compartment has 15 chains birds must flow through before migrating away; the rate of flow is equivalent to 15 chains / (135 days – *q_B_*) or 0.192 birds per day. Similarly, the rate susceptible adults transition into the overwinter susceptible compartment (*γ_A_*) depends on when they arrive and is the inverse of the duration of the breeding season, 135 days (0.0074 birds per day x 25 chains = 0.185 birds per day). Because chronic infections vary in how long they persist, we set the rates chronically infectious first year (*σ_J_*) and adult (*σ_A_*) birds transition through the chronically infectious chains (4 chains) to eventually become latently infected overwintering birds to a value of 0.230 birds per day (divided by 4 chains = 0.058 birds per day) to ensure that all chronically infectious birds entered the overwinter, latently infected compartment at the end of the breeding season.

***Black fly compartmental transition parameters***

We parameterized the rate an exposed black fly becomes infectious (*λ_F_*) on the average prepatent period (5-6 days) of black flies (*Prosimulium decemarticulatum* Twinn, *S. aureum* Fries, *S. quebecense* Twinn, *S. latipes* Meigen*,* and *Cnephia ornithophilia*) naturally infected with *L. fringillinarum* . We assumed the average rate of *λ_F_* to be approximately 0.142 flies per day (prepatent period of 7 days) because average temperatures can be lower at our high elevation sites than laboratory infection conditions. We assumed a uniform distribution for this parameter with a minimum and maximum values ranging from 0.0714-0.246 flies per day. Finally, there is no evidence that black flies clear *Leucocytozoon* infection throughout their relatively short lifespan , so to ensure that there were no overwintering black flies we incorporated four chains into the acutely infectious compartment that black flies flow through until they die of background induced mortality. The rate of flow (*σ_F_*) is model programmed and set to 0.055 black flies per day, and it was also not included in our sensitivity / uncertainty analyses.

***Nestling hatch and black fly emergence functions***

For all parameters in both the nestling hatch and black fly emergence functions, we assumed uniform distributions for the uncertainty analysis. Based on reproductive data collected from our field sites on a breeding population of Mountain White-crowned Sparrows (*Zonotrichia leucophrys oriantha*), we assumed the model average number of eggs produced per female per day (*A_B_*) to be approximately 0.148 eggs per female per day (see supplementary information A for more details) with values ranging from 0.129-0.167 eggs per female per day. The model also includes the variable *c_B_* (support of the nestling hatch function) a parameter that determines the width of the nestling hatch function. We assumed a possible range of 150-250, and an average model value of 200. Finally, we set the average date of the peak of the nestling hatch function (*q_B_*) to occur on day 57 with a possible range of dates spanning from day 30-85.

The black fly emergence function, like the nestling hatch function, was parameterized off of field collections of black flies on our field sites (see supplementary information A for more details). We set the peak of black fly emergence to an average model value of 70 emerging black flies per day, and we assumed possible values for this parameter could range from 50-90 emerging black flies per day. We set the support of the black fly emergence function (controls width of emergence function) to an average value of 600, with a possible range of values from 500-700. Finally, we set the date of peak black fly emergence to occur on day 66, with possible values ranging from day 45-87.

***Death parameters***

We had three parameters modeling avian death: the natural death rate of nestlings (*d_1_*), the natural death rate of feathered birds (*d_2_*), and the parasite-induced death rate of acutely infectious birds (*d_3_*). We parameterized the natural death rate of nestlings and feathered birds from a series of Mountain White-crowned Sparrow populations, including data collected on our field sites . We assumed that nestlings and feathered birds died at an average rate of 0.032 nestlings per day and 0.000616 birds per day, respectively. We assumed a gamma distribution with a possible range of values from 0.0093-0.033 nestlings dying per day and 0.0023-0.0018 birds dying per day, respectively. Due to significant overwinter mortality, especially for first year birds, we adjusted the number of birds returning to breed in the subsequent field season by allowing 20% of first year (*x_J_*) and 80% of adult birds (*x_A_*) to return . We did not allow these parameters to vary, so we did not include them in the uncertainty analysis. We parameterized the death rate of acutely infectious birds (*d_3_*) from natural infections of domestic ducks (*A. platyrhynchos*) with relatively virulent *L. simondi* in northern Michigan. We set the average value of *d_3_* to be 0.001 birds dying per day However, the death rate of acutely infectious birds in the field has not been clearly determined, and infections can vary in virulence. Thus, we assumed a gamma distribution for this parameter, with possible values ranging from 0.0009 birds dying per day (low virulence) to 0.5 birds dying per day (high virulence). Finally, we assumed that all black flies, regardless of infection status, experienced a natural background mortality of 0.167 black flies dying per day following a gamma distribution on 1/*d_4_* with a range of possible values spanning 0.033-0.815 black flies dying per day.

1 Briggs NT (1960) A comparison of *Leucocytozoon simondi* in pekin and muscovy ducklings. Proceedings of the Helminthological Society 27: 151-156.

2 Samuel MD *et al.* (2011) The dynamics, transmission, and population impacts of avian malaria in native Hawaiian birds: a modeling approach. Ecological Applications 21: 2960-2973.

3 Komar N *et al.* (2003) Experimental infection of north American birds with the New York 1999 strain of West Nile virus. Emerging Infectious Diseases 9: 311-322.

4 Roller NF, Desser SS (1973) Diurnal periodicity in peripheral parasitemias in duckling (*Anas boschas*) infected with *Leucocytozoon simondi* Mathis and Leger. Canadian Journal of Zoology-Revue Canadienne De Zoologie 51: 1-9, doi:10.1139/z73-001.

5 Valkiunas G (2005) Avian malaria parasites and other haemosporidia. New York: CRC Press.

6 Mackinnon MJ, Read AF (2004) Virulence in malaria: an evolutionary viewpoint. Philosophical Transactions Of The Royal Society Of London Series B-Biological Sciences 359: 965-986.

7 Mackinnon MJ, Read AF (2003) The effects of host immunity on virulence-transmissibility relationships in the rodent malaria parasite Plasmodium chabaudi. Parasitology 126: 103-112.

8 Khan RA, Fallis AM (1970) Life cycles of *Leucocytozoon dubreuili* Mathis and Leger, 1911 and *Leucocytozoon fringillinarum* Woodcock, 1910 (Haemosporidia: Leucocytozoidae). Journal of Protozoology 17: 642-658.

9 Khan RA, Fallis AM (1970) Relapses in birds infected with species of *Leucocytozoon*. Canadian Journal of Zoology-Revue Canadienne De Zoologie 48: 451-455.

10 Morton ML (2002) The Mountain White-crowned Sparrow: migration and reproduction at high altitude. Camarillo, CA: Cooper Ornithological Society.

11 Baker MC, Mewaldt LR, Stewart RM (1981) Demography of White-crowned Sparrows (*Zonotrichia leucophrys nuttalli*). Ecology 62: 636-644.

12 Chilton G, Baker MC, Barrentine CD, Cunningham MA (1995) White-crowned Sparrow (*Zonotrichia leucophrys*). Washington, D. C.

13 Chernin E (1952) The epizootiology of *Leucocytozoon simondi* infections in domestic ducks in northern Michigan. American Journal of Hygiene 56: 39-57.

14 Adler PH (2004) The black flies (Simuliidae) of North America. Ithica, NY: Cornell University Press in association with the Royal Ontario Museum.
