# Supplementary material for: A Transmission Model for the Ecology of an Avian Blood Parasite in a Temperate Ecosystem
Source: PLoS One. 2013 Sep 20;8(9):e76126. doi: 10.1371/journal.pone.0076126 (PMC3779181; doi:10.1371/journal.pone.0076126)

**Text S5**

**Parameter Plots**

*Parasite prevalence is sensitive to the following avian parameters:*

1. *γ_A_*, the rate susceptible adult birds flow into the susceptible, overwinter stage


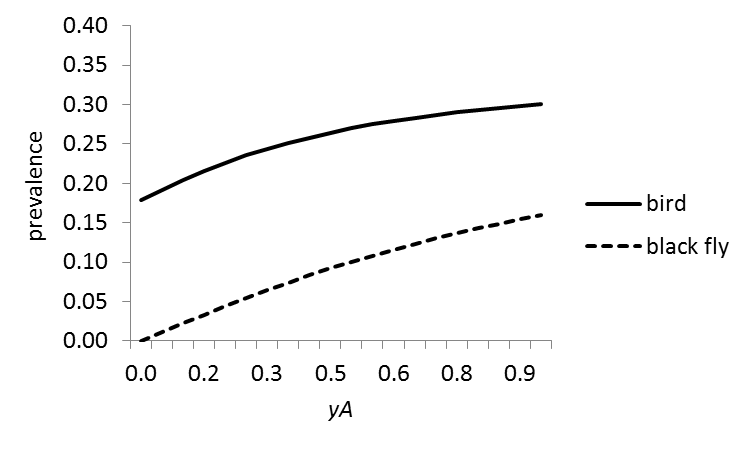


1. *b_F_*, the probability of transmission from an infectious black fly to a susceptible feathered bird


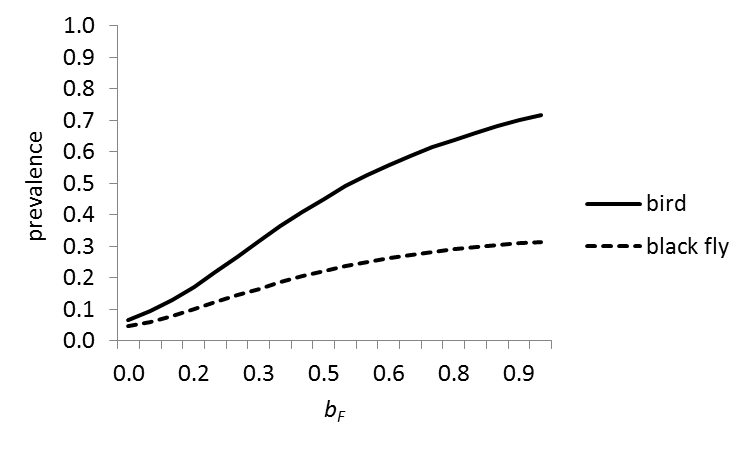


1. *δ_R_*, the rate relapsing infectious adults flow into the latently infected, overwinter stage


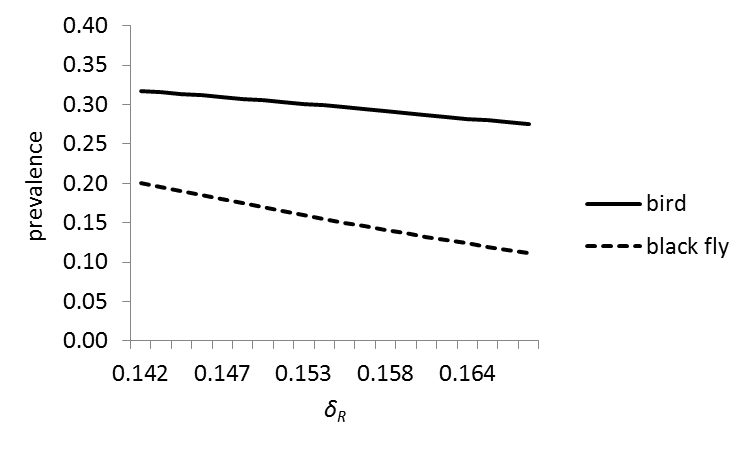


1. *A_B_*, the peak number of nestlings hatching per day


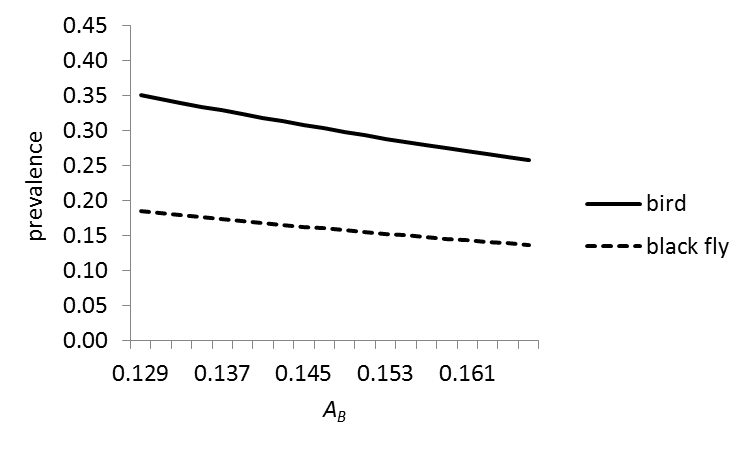


1. *c_B_*, the duration of the nestling hatch period


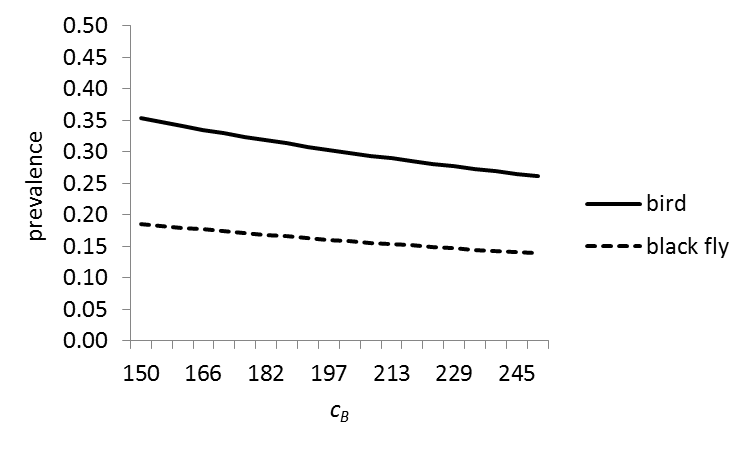


*Parasite prevalence is sensitive to the following black fly parameters:*

1. *r*, the number of bites female black flies take per day


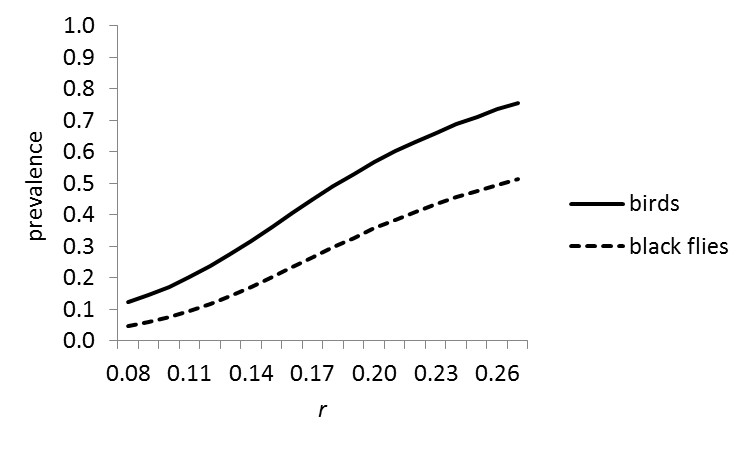


1. *b_R_*, the probability of successful transmission from a relapsing infectious bird to a susceptible black fly


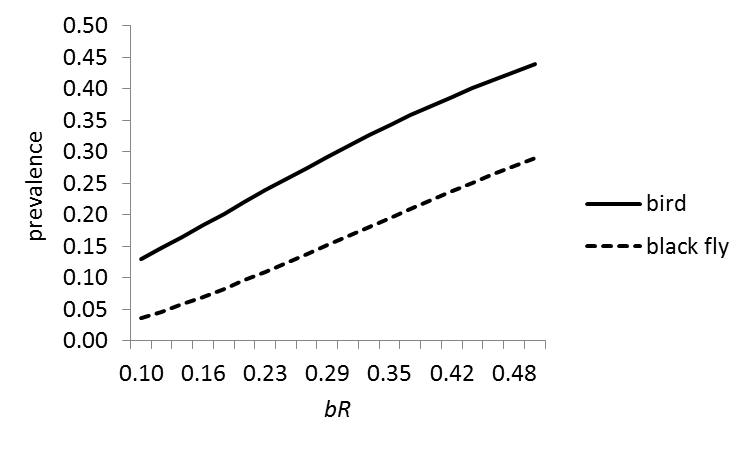


1. *d_4_*, the natural death rate of susceptible, host-seeking black flies


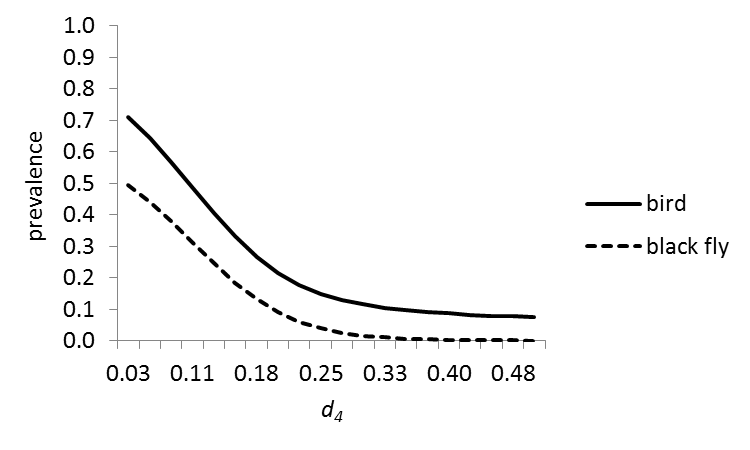


1. *q_F_*, the date of peak black fly emergence during the transmission season


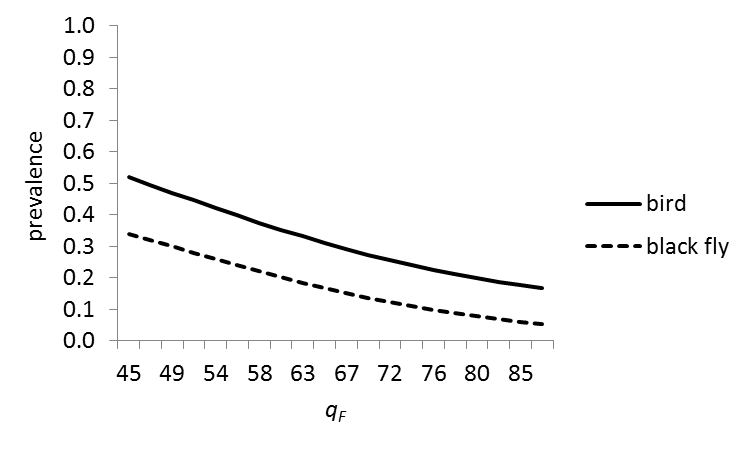


1. *c_F_*, the duration of the black fly emergence period


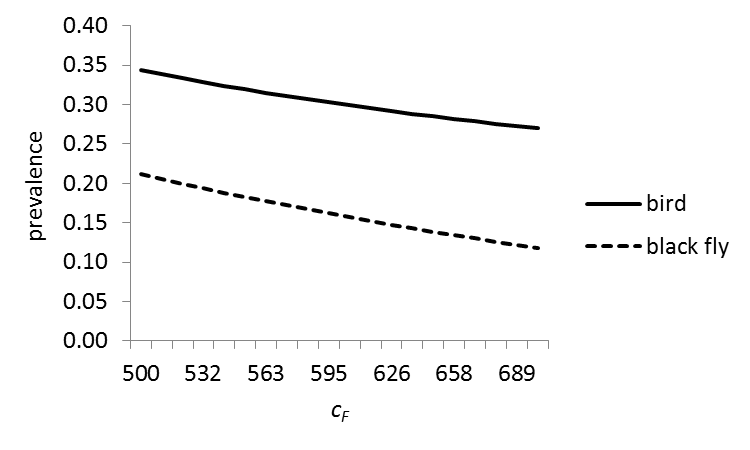

Supplement: Text S5 — Parameter plots of the parameters avian and black fly infection prevalence were most sensitive to as indicated in the sensitivity analysis. (DOC) [file pone.0076126.s005.doc]
